# Report of Committee on Medical Societies and Colleges, on the Establishment of an Asylum and a School for Idiots

**Published:** 1847-04

**Authors:** 


*Report of Committee on Medical Societies and Colleges, on the establishment of an asylum and a school for idiots.*—It will be recollected by our readers that at the session of our Legislature preceding the present, the committee on medical schools, and colleges, of which Dr. F. F. Backus is chairman, reported in favour of instituting an asylum for idiots on the plan of the institutions which are now in successful operation in Europe. The same committee the present session, Dr. B. being still the chairman, urged the project in an earnest matter upon the attention and action of the Legislature now in session. The state society at its late meeting passed a resolution commending it to the favorable consideration of our legislators. We sincerely hope that the philantrophic efforts of Dr. B. and his associates of the committee will prevail.

